# Injuries of Parturition

**Published:** 1898-05

**Authors:** 


### Injuries of Parturition.

A. H. Tuttle, in a paper read before the American Medical Association (*Journal Am. Med. Assoc.*), concludes that the obstetrician who today boasts that in his practice he has no lacerations of the cervix and perineum, proclaims to the world his ignorance of this branch of his profession. It is as much the duty of the obstetrician to his patient to see that she is properly repaired as to see her safely delivered. The public should be educated to the fact that injuries to the soft parts of the parturient canal are often inevitable, and that such injuries only cast a reflection on the professional attendant when their after-treatment is neglected. A physician in whose practice a laceration of the parturient canal has occurred can protect himself in no better way from a legal attack for alleged malpractice than by calling in assistants, who serve as witnesses, within twenty-four hours of the time of the accident, and repairing properly, or offering to repair, the injuries which his patient has sustained. There is no better time to repair the injuries of parturition than within twenty-four hours of the time of the occurrence; such immediate repair of the injury will often hasten convalescence and prevent in many cases severe pelvic complications. The obstetrician should be educated to do this part of his work in a more careful and thorough manner. The injuries already sustained by a multipara in previous labors should be an incentive rather than an objection to the careful treatment of the lacerations of her present delivery, because a repair of her total injuries can be accomplished at the same time.—*The Medical Standard.*

